# Identification of D-Limonene Metabolites by LC-HRMS: An Exploratory Metabolic Switching Approach in a Mouse Model of Diet-Induced Obesity

**DOI:** 10.3390/metabo12121246

**Published:** 2022-12-09

**Authors:** José Fernando Rinaldi de Alvarenga, Caroline Lei Preti, Lara Santos Martins, Guilherme Noronha Hernandez, Brunna Genaro, Bruna Lamesa Costa, Caroline Gieseler Dias, Eduardo Purgatto, Jarlei Fiamoncini

**Affiliations:** 1 Human Nutrition Unit, Department of Food and Drug, University of Parma, 43125 Parma, Italy; 2 Food Research Center (FoRC), University of São Paulo, Rua do Lago 250, São Paulo 05508-080, Brazil; 3 Department of Food Science and Experimental Nutrition, Faculty of Pharmaceutical Sciences, University of São Paulo, Avenida Prof. Lineu Prestes 580, São Paulo 05508-000, Brazil

**Keywords:** xenobiotic metabolism, monoterpenes activity, high-resolution mass spectrometer

## Abstract

Metabolic switching has been raised as an important phenomenon to be studied in relation to xenobiotic metabolites, since the dose of the exposure determines the formation of metabolites and their bioactivity. Limonene is a monoterpene mostly found in citrus fruits with health activity, and its phase II metabolites and activity are still not clear. The aim of this work was to evaluate the effects of D-limonene in the development of diet-induced obesity in mice and to investigate metabolites that could be generated in a study assessing different doses of supplementation. Animals were induced to obesity and supplemented with 0.1% or 0.8% D-limonene added to the feed. Limonene phase I and II metabolites were identified in liver and urine by LC-ESI-qToF-MS/MS. To the best of our knowledge, in this study three new phase I metabolites and ten different phase II metabolites were first attributed to D-limonene. Supplementation with 0.1% D-limonene was associated with lower weight gain and a trend to lower accumulation of adipose tissue deposits. The metabolites limonene-8,9-diol, perillic acid and perillic acid-8,9-diol should be explored in future research as anti-obesogenic agents as they were the metabolites most abundant in the urine of mice that received 0.1% D-limonene in their feed.

## 1. Introduction

Monoterpenes are volatile phytochemicals produced by the secondary metabolism of plants involved in the perception of aroma and flavor of fruits and vegetables [1,2]. These compounds have been used in cuisine and food preservation, as well as in therapeutic applications, due to their antimicrobial, analgesic, and anti-inflammatory effects [3,4,5]. Recently, anti-obesity and anti-diabetic activities have been attributed to monoterpenes, suggesting that these compounds can also modulate energy metabolism in mammals [6,7]. Limonene is one of the most common monoterpenes in nature and a major constituent of citrus essential oil. Limonene has a lemon-like odor and is widely used as a flavor and fragrance additive in juices, candies, soft drinks, and ice creams [8]. After its consumption, limonene is rapidly absorbed, distributed, and metabolized, offering low toxicity to humans [8,9]. Studies in animal and different cell models have reported hypolipemic and hypoglycemic effects of limonene, which also stimulates beta-oxidation of fatty acids and contributes to reduced weight gain [10,11,12,13,14]. Sub-chronic and chronic studies that use a high dose of limonene or other phytochemicals can be misleading as they could cause a saturation of the pathway involved in the phytochemical’s metabolism with two likely consequences: first, the excessive substrate could cause the saturation of the enzymes responsible for the xenobiotic metabolization, leading to the generation of new phase I metabolites; second, the lack of appropriate co-factors could favor phase II conjugation with sulfate and amino acids. The use of alternative metabolic pathways or the switch to a new one could generate new metabolites, which would not be generated if the animal was exposed to lower doses of the compound. This phenomenon is known as “dose–response metabolism” or “metabolic switching”, and the results of studies in which this effect is observed may be misleading or irrelevant to humans exposed to low doses of the compound in question [2,6,15,16]. Therefore, to better understand limonene bioactivity, it is necessary to identify the main metabolites produced, which could be responsible for the biological effects. The aim of this work was to evaluate the effects of D-limonene in the development of diet-induced obesity in mice and to investigate metabolites that could be generated in a study with different doses.

## 2. Materials and Methods

### 2.1. Chemicals and Standards

Methanol and acetonitrile LC-MS grade were bought from Merck (Darmstadt, Germany). Ultra-pure water was purified by a Milli-Q system (Millipore, Bedford, MA, USA). Formic acid LC-MS grade was purchased from Sigma Aldrich. (R)-(+)-Limonene and myrcene were bought from Fluka (Morristown, NJ, USA) and deuterated internal standard (IS) cholic acid-2,2,4,4-D4 was obtained from CDN-Isotopes (Point-Clair, QC, Canada).

### 2.2. Animal Study

The experiments were performed in compliance with the Animal Experimentation Ethics Committee (CEUA) of the School of Pharmaceutical Sciences (FCF) of the University of Sao Paulo (USP), Brazil (protocol 576). In this study, male 11-week-old C57/Bl6 mice were obtained from the facility for animal production of FCF/USP. The animals were acclimated for 1 week to the environment of the facility for animal and the chemically defined feed. After the adaptation period, mice were divided into four groups, one maintaining a normolipidemic diet and the other three on a high-fat diet. From these, two groups received feed supplemented with 0.1 and 0.8% D-limonene. The animals received diet and water ad libitum and were kept at 22 °C with a 12 h light-dark cycle. Immediately before the start of the supplementation and after 6 weeks of supplementation, samples of the animals’ urine were collected.

Commercial diets were provided by Rhoster (Araçoiaba da Serra, SP, Brazil). Normolipidic diet contained 13.45% protein, 68.92% carbohydrate, and 3.82% fat. High-fat diets contained 60% of energy in lipids, including 23.5% protein, 27.3% carbohydrate, and 34.3% fat (37% saturated, 47% monounsaturated, and 16% polyunsaturated), and were supplemented with 0.1% and 0.8% orange essential oil containing 96% D-limonene (Table 1).

### 2.3. Analysis of D-Limonene in Essential Oil and Feed

#### 2.3.1. Analysis of Essential Oil Rich in D-Limonene GC-MS

Orange essential oil rich in D-limonene was kindly supplied by Citroflavor (Catanduva, SP, Brazil). The purity of the essential oil was verified by GC-MS, Agilent CG 6850 coupled to Agilent mass spectrometer 5975C. The compounds were separated using a HP-5MS column (30 m × 0.25 mm × 0.25 µm) and the CG inlet was set at 250 °C using a split mode, quadrupole at 180 °C and ion source at 250 °C. Helium was used as a carrier gas with a flow rate of 1.00 mL·min^−1^. The oven programmed at 40 °C held for 5 min, ramp rate of 6 °C·min^−1^ until 100 °C and then 3 °C·min^−1^ up to 205 °C. The identification was performed by comparing fragmentation pattern with NIST library.

#### 2.3.2. Sample Extraction

Feed pellets (normolipidic diet) were homogenized in a blender using a pulse system to avoid heat and were then sieved to obtain particles smaller than 0.2 mm. Samples (1.00 g) were spiked with 100 µL of internal standard (IS) and extracted with 2.5 mL of chloroform, vortexed for 30 s, and centrifuged using 10,000 rpm for 10 min at 4 °C. The supernatant was collected, and a re-extraction was performed. The supernatants were combined and completed up to 5 mL in a volumetric flask and immediately analyzed by GC-FID.

#### 2.3.3. Quantification of D-Limonene by GC-FID

A Shimadzu 2010 (Kyoto, Japan) was used for the CG-FID analysis. The capillary column was DB-5 (30 m × 0.25 mm × 0.25 µm). The CG inlet was set at 250 °C using a splitless mode, and FID temperature was 250 °C. The hydrogen carrier flow rate was 1.50 mL·min^−1^. The oven programmed at 40 °C held for 3 min, ramp rate of 3 °C·min^−1^ until 80 °C and then 10 °C·min^−1^ up to 230 °C and held for 10 min. The internal standard calibration was used for quantification. Calibration curves for D-limonene were constructed using myrcene (not identified in the essential oil) as internal standard (2.4 ppm) with 7 points ranging from 0.0085 to 4.29 ppm, and the results were expressed in g of limonene per 100 g^−1^ of fed.

### 2.4. Identification of Limonene Metabolites by LC-ESI-qToF-MS/MS

#### 2.4.1. Sample Extraction

Liver samples (10 mg) were spiked with 20 µL of 2 uM of IS solution, extracted with 400 µL of cold methanol, vortexed at 600 rpm for 10 min at 4 °C, and then centrifuged at 12,000 rpm for 15 min at 4 °C. The supernatant was transferred to clean tubes and a re-extraction was performed. The supernatants were combined and evaporated until dry using a vacuum concentrator (Concentrator plus, Eppendorf, Germany). The residue was reconstituted with 200 µL of methanol:water (1:1, *v*/*v*), filtered using a 0.22 µm PTFE filter into a HPLC vial and stored at −80 °C until analysis.

An amount of 30 µL of urine samples were added with 70 µL of 0.286 uM of IS, prepared in methanol, vortexed for 30 s, and centrifuged at 12,000 rpm for 10 min at 4 °C. The supernatant was collected and transferred to HPLC vials and analyzed.

#### 2.4.2. Identification of D-Limonene Metabolites by LC-HRMS

Limonene metabolites identification were performed by LC-MS. A UPLC system (Nexera XR model) equipped with a binary pump, autosampler, and oven from Shimatzu (Kyoto, Japan), with a Kinetex EVO C18 column (100 mm × 2.1 mm) 1.7 µm (Phenomenex, Torrance, CA, USA) was used. The injection volume was 10 µL, the samples were maintained at 8 °C and the column at 40 °C. The separation of limonene metabolites was carried out with water 0.1% of formic acid (phase A) and acetonitrile 0.1% of formic acid (phase B). A gradient elution was applied as follows: 0 min, 5% B; 12 min, 100% B; 13 min, 100% B; 15 min, 5% B, with a flow rate of 400 µL/min. The MS analysis was performed using a qToF analyzer (Compact, Bruker Daltonics, Bremen, Germany) equipped with an ESI source and operated in negative and positive ion mode. A capillary voltage of +/−4.5 KV, end plate offset of 500 V, nebulizer gas pressure of 28.0 psi, dry gas flow of 10 L/min, and dry temperature of 200 °C were applied. Data were collected in profile mode in the range of 50–1000 *m*/*z*. The samples were also analyzed in the dependent data mode in which the highest intensity ions were selected to perform the product ion analysis to identify possible metabolites. To ensure data quality, sodium formate was used as a reference ion to calibrate the equipment. Sample randomization was performed to avoid the injection order effect, and a quality control, containing an equal mixture of all samples, was used to ensure the quality of the data from LC-ESI-qToF-MS/MS. DataAnalysis and QuantAnalysis software from Bruker were used for identification and peak area extraction, respectively.

Identification was performed by the detected pseudo-molecular ion that must differ from the exact mass of proposed molecular formula in a maximum value of 10 ppm, and isotopic pattern was checked with theorical isotope profile. Identification was confirmed by MS/MS experiments and comparison with databases and literature. Metabolites were classified according to metabolomics guidelines [17].

### 2.5. Creatinine Analysis

Creatinine content in urine was determined using commercial kit Creatinine K from Labtest (Vista Alegre, MG, Brazil) by colorimetric assay by alkaline picrate Jaffé reaction, which was measured in spectrophotometer Biotech Synergy H1 (BioTek Instruments, Winooski, VT, USA).

### 2.6. Statistical Analysis

The Shapiro–Wilk test was used to check data normality. The statistical differences were analyzed by *t*-test and Wilcoxon test for comparisons between two groups and ANOVA followed by Tukey test and Dunn’s Kruskal–Wallis for multiple comparisons according to data normality. A two-way ANOVA was performed for multiple comparison with Fisher’s LSD test in the time-course data. Statistical analysis was performed by R v3.4.4. and GraphPad Prism 8 software (San Diego, CA, USA).

## 3. Results

### 3.1. Effect of D-Limonene-Supplemented Diet on Body Weight Gain and Feed Intake

Animals that received a high-fat diet had greater weight gain when compared to the normolipidic group. The HL0.1 group showed a trend of lower weight gain during the protocol, and at the end of six weeks it showed a statistical difference from the HL, albeit without differences from the HL0.8 (Supplemental Material Figure S1C). There was no difference in feed intake, regardless of supplementation, indicating that there was no rejection of the feed due to the limonene scent (Supplemental Material Figure S1B). A lower energy efficiency was observed for HL0.1 when compared to the other high-fat groups, but there was no statistical difference with any experimental group, indicating a lower obesogenic effect of the diet when supplemented with 0.1% D-limonene.

Six weeks of D-limonene-supplemented diet were able to modify the organs weight in high-fat-induced obese mice. A trend of lower epididymal, retroperitoneal, and subcutaneous fat accumulation was observed for the HL0.1 group, differing statistically from the HL0.8 but not from the HL. However, all high-fat diet groups showed a statistical difference from the NL group, except for HL0.1, indicating a proximity to the normolipidic diet phenotype (Supplemental Material Figure S1C,D,G–I). No differences were found in the weight of the livers, regardless of the treatment (Supplemental Material Figure S1F).

### 3.2. Quantification of D-Limonene Intake

GC-MS analysis of essential oil composition from orange peel revealed the presence of eight monoterpenes and an aldehyde (Table 1). Limonene was the major compound in the essential oil, representing 96% of the relative area of the chromatogram. The feeds used in the experiment were analyzed for their D-limonene content in order to identify losses during the feed production process and animal feeding (feed remained in the appropriate compartment in the mice cage for 24–48 h). The high-fat diet theoretically containing 0.1% D-limonene showed a content of 0.10 g/100 g of feed without losses during processing, while the diet added with 0.8% D-limonene had a content of 0.67 g/100 g, with a loss of 16.25%. Due to the volatility of D-limonene, a stability experiment was performed to verify the loss of this molecule exposed to 30 °C in 24 h, showing a maximum loss of 33% in relation to the initial content (data not shown).

The actual D-limonene intake by the animals was calculated based on the D-limonene content of the feed, showing an average consumption of 2.37 ± 0.13 mg/day for the animals from the HL0.1 group and 17.55 ± 1.04 mg/day for the animals from the HL0.8 group during the six weeks of experimental protocol. D-limonene intake in mice from the HL0.1 group was on average 90 ± 10 mg/kg, while in mice from the HL0.8 group, it reached 580 ± 60 mg/kg [18,19].

### 3.3. Identification of D-Limonene Metabolites

**Figure 1:**
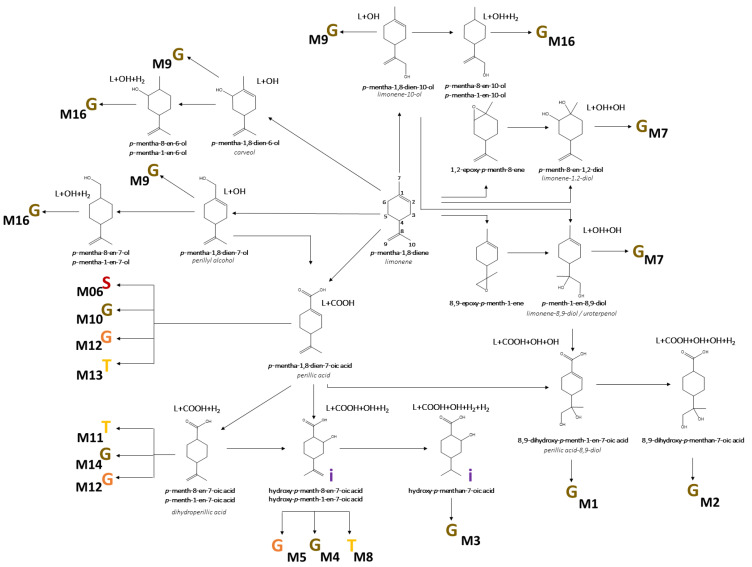
Metabolic map of possible metabolism pathways used for D-limonene excretion. Phase II metabolites were indicated as glucuronide (brown G), sulfate (S), glycine (orange G), and taurine (T). Isomers were indicated by i.

A literature survey of the main metabolites derived from limonene was previously carried out by our group to corroborate identification [6]. Furthermore, we searched for possible metabolization reactions described for xenobiotics and in silico tools to determine new pathways for phase I reactions and phase II conjugations [20,21]. Metabolites were determined in samples from the groups supplemented with D-limonene, and their absence was verified in the non-supplemented groups. The analysis of liver and urine in the search of D-limonene metabolites tentatively identified 16 different metabolites in urine and 3 in the liver (Table 2). The biotransformation of D-limonene occurs by phase I and II reactions, mainly by processes of hydroxylation, carboxylation, and dehydrogenation, mostly followed by conjugation with glucuronic acid. Samples from animals supplemented with limonene were compared to samples from the HL group to exclude possibly endogenous metabolites (Figure 1).

The most polar metabolite (first eluted from the reversed-phase column) was identified as M1, with a retention time (rt) of 1.89 min, *m*/*z* of 375.1304, and the molecular formula (MF) C_16_H_23_O_10_. The MS/MS experiment revealed the presence of a glucuronide moiety with the fragments *m*/*z* of 113.0224, 93.0353, and 85.0291. Due to the loss of water from the parent ion, the fragments showed water loss by 357.1104 [M-H-H_2_O], 183.1088 [M-H-H_2_O-O-glucuronide], and 172.9903 [M-H-H_2_O-183.1088], corresponding to glucuronide moiety. Therefore, the aglycon form of the molecule would have a mass of 199.0984, which compared to limonene had a gain of a carboxylic acid group and two hydroxylations. This aglycone could be labeled as 8,9-dihydroxy-p-menth-1-en-7-oic acid, also named perillic acid-8,9-diol, described in the literature [22,23]. For that, M1 was tentatively identified as limonene+COOH+OH+OH glucuronide. M2 (*m*/*z* 377.1460) was eluted at 2.01 min and the mass difference between M1 and M2 showed a hydrogenation process, probably in the benzene ring of the limonene. The MS/MS analysis showed the loss of the glucuronide moiety, with an *m*/*z* of 201.1180 [M-H-glucuronide] and *m*/*z* 175.0236 [glucuronide], and the fragmentation pattern of glucuronic acid with an *m*/*z* of 113.0251, 93.0344, 85.0293, and 75.0087. M2 was tentatively identified as limonene+COOH+OH+OH+H_2_ glucuronide.

Metabolites M3, M4, M5, and M8 represent compounds that have undergone carboxylation and hydroxylation reactions. M3 has an *m*/*z* of 361.1504 and rt of 2.83 min with a possible MF of C_16_O_25_O_9_, which would indicate an error of −0.1 ppm. The fragmentation pattern revealed the presence of a glucuronic moiety, by the ion *m*/*z* of 175.0256, together with other fragments already described. The presence of the fragment *m*/*z* of 185.1181 [M-H-glucuronide] indicates that in the other part of the molecule, compared with limonene, the presence of a carboxylic acid group, a hydroxyl, and two reductions reaction were found. M3 was tentatively identified as limonene+COOH+OH+H_2_+H_2_ glucuronide and not previously reported in the literature. The M4 metabolite showed three isomers, M4-I, M4-II, and M4-III, which showed the same fragmentation pattern at a different rt of 2.95, 3.18, and 3.57 min, respectively. The parent ion detected had an *m*/*z* of 359.1344 (error 1.0 ppm) for M4-I, 359.1345 (error 0.8 ppm) for M4-II, and 359.1326 (error 6.0 ppm) for M4-III. The difference in mass for M3 to M4 metabolite showed the presence of a doubled bound that was not lost in the metabolization process, indicated by an *m*/*z* of 183.10. Furthermore, the pattern of glucuronide fragmentation indicates the presence of a glucuronide moiety, corroborating the identification of limonene+COOH+OH+H_2_ glucuronide as a phase I metabolite not yet described by literature. The literature reported the presence of a phase I metabolite as 2-hydroxy-p-menth-8-en-7-oic acid, with a theorical mass of 184.1099, which helps to corroborate the identification of M3 and M4 [22]. Limonene+COOH+OH+H_2_ was also found conjugated to glycine, as M5-I and M5-II, and conjugated to taurine, as M8. M5 metabolites were found with an *m*/*z* of 240.1224 (error 7.2 ppm) and an rt of 3.41 min and with an *m*/*z* of 240.1229 (error 5.3 ppm) and an rt of 3.62 min, corresponding to M5-I and M5-II, respectively. MS/MS experiments revealed the presence of the high intensity fragment *m*/*z* of 74.0242, corresponding to glycine. The strong ionization of the glycine moiety makes it difficult to visualize other fragments, but a fragment *m*/*z* of 178.12 was detected, which could correspond to the parent ion with the loss of a CO_2_ and a H_2_O molecule [M-H-H_2_O-CO_2_]. The difference in the loss of glycine portion shows a mass of 166.0985 [C_10_H_14_O_2_], which corresponds to the breakage of the amide bond, indicating the phase I metabolite loses a hydroxyl group. M8 was detected with an *m*/*z* of 290.1054 (error 4.6 ppm) at 3.92 min with an MF C_12_H_20_NO_5_S. Molecule fragmentation revealed that an *m*/*z* of 124.0067 [M-H-C_2_H_6_NO_3_S] corresponds to a taurine moiety and an *m*/*z* of 79.9568 represents a sulfate, which is part of the taurine molecule. The difference between the parent ion and the taurine molecule also has a mass of 166.0987 [C_10_H_14_O_2_], following the same logic for the glycine conjugate.

**Figure 2:**
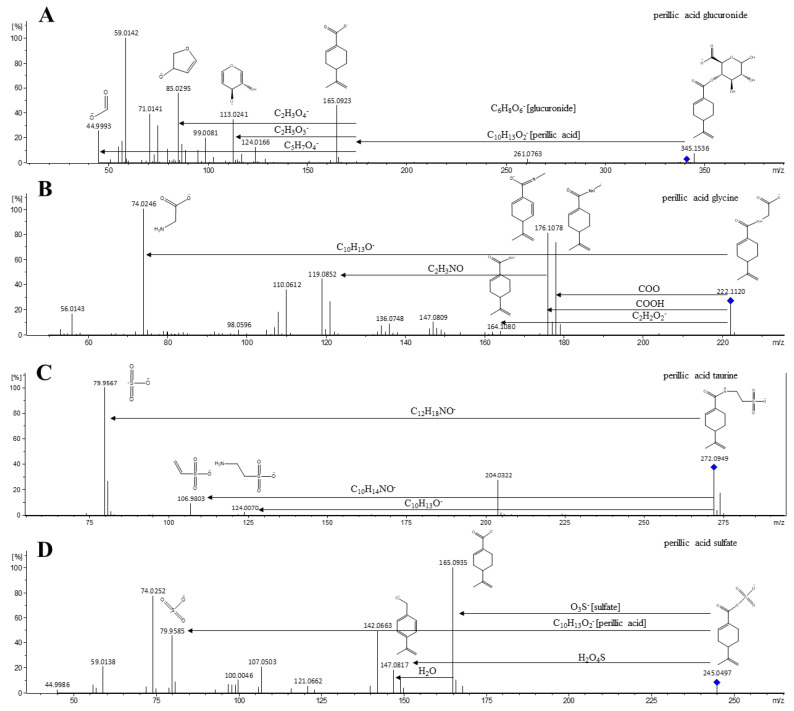
Fragmentation map of different phase II metabolites from perillic acid. (**A**) perillic acid glucuronide; (**B**) perillic acid glycine; (**C**) perillic acid taurine; and (**D**) perillic acid sulfate.

One of the main metabolites derived from limonene is perillic acid, formed after the introduction of a carboxylic group in the limonene molecule [22,23,24,25,26,27]. The metabolites M6, M10, M12, and M13 represent this phase I biotransformation with different forms of phase II conjugation. M6 was detected at rt 3.62 min, with an *m*/*z* of 245.0490 (error −0.3 ppm) and a theorical MF of C_10_H_13_O_5_S. The MS/MS fragmentation revealed the presence of an *m*/*z* of 79.9569 that corresponds to a sulfate and the complementation moiety of 165.0919 [M-H-sulfate] being limonene+COOH. M10 showed an *m*/*z* of 341.1243 (error −0.3 ppm) with an MF of C_16_H_21_O_8_ at 449 min. The detection of an *m*/*z* of 175.0230 and fragments of 113.0241, 99.0081, and 85.0295 revealed the presence of a glucuronic group, and the breakage of the ester bond led to the detection of an *m*/*z* of 165.0923, corresponding to limonene+COOH. Limonene+COOH glucuronide, identified as perillic acid glucuronide, has been previously described [22,26]. Metabolites M12 and M13 represents conjugations with glycine and taurine, respectively, by an amine bond. M12 was detected with an *m*/*z* of 222.1134 (error 0.8 ppm), at 4.63 min with an MF of C_12_H_16_NO_3_. The fragmentation reveals an *m*/*z* of 178.1218 [M-H-CO_2_] and 176.1078 [M-H-HCOOH], which were losses possibly attributed to parts of the glycine molecule and 121.1019 [M-H-C_3_H_2_NO_3_], which could represent the glycine attached with the carboxylic group. In addition, glycine was detected with a high-intensity *m*/*z* of 74.0246. Limonene+COOH conjugated to glycine was reported [22]. M13 has an *m*/*z* of 272.0949 (error 5 ppm), rt = 4.66 min, and fragments *m*/*z* values of 204.0322 [M-H-C_5_H_8_] and 164.1039 [M-H-C_2_H_3_O_3_S], and its complement was found to be at 106.9803 [C_2_H_3_O_3_S]. Furthermore, the presence of *m*/*z* values of 124.0070 and 79.9567, identified as taurine and its sulfate moiety, corroborate the identification. M13 was tentatively identified as limonene+COOH conjugated with taurine. The fragmentation of conjugates with amide bound is different from ester bonds, and the difference in the mass of glycine and taurine in their respective metabolites is 148.08, indicating the presence of limonene+COOH loses a hydroxyl group. The fragmentation pattern of different phase II metabolites from perillic acid were reported in Figure 2.

The biotransformation of limonene into its carboxylated derivative, perillic acid, favors the production of dihydroperillic acid, through the hydrogenation process [25]. Metabolites M11, M14, and M15 were identified as limonene+COOH+H_2_ conjugated with taurine, glucuronide, and glycine, respectively. M14 was detected with an *m*/*z* of 343.1389 (error 1.3 ppm) and a theorical MF of C_16_H_23_O_8_, and the identification was corroborated by the presence of a fragment *m*/*z* of 175.0235 and its complements, indicating a glucuronidation and the other moiety with an *m*/*z* of 167.1069 that corresponded to limonene+COOH+H_2_. M11, with an *m*/*z* of 274.1101 (error 6.2 ppm), and M15, with an *m*/*z* of 224.1177 (error 6.7 ppm), were identified as those conjugated with taurine and glycine, respectively. Both showed similar fragmentation patterns to the limonene+COOH reported for M12 and M13 metabolites.

The production of hydroxylated and dehydroxylated metabolites were also previously described for limonene [22,23,24,26,27,28,29,30]. Three different isomers were found for the M7 metabolite, with an *m*/*z* of 345.1550 (error 1.4 ppm) for M7-I, an *m*/*z* of 345.1548 (error 2.1 ppm) for M7-II, an *m*/*z* of 345.1538 (error 5.0 ppm) for M7-III, and a theorical MF of C_16_H_25_O_8_. Only M7-I and M7-II showed fragmentation results in which the presence of a glucuronic group was revealed by the aglycone portion with an *m*/*z* of 169.12. The addition of two hydroxyls to the limonene molecule were verified, resulting in a metabolite that can be identified as limonene+OH+OH glucuronide. Previous reports indicate that the presence of uroterpenol (p-menth-1-en-8,9-diol) [22,23,24,26,28] and limonene-1,2-diol (p-menth-1-en-1,2-diol) [28] could possibly be results of the epoxidation process by hepatic enzymes. Furthermore, there is a favoring of dihydroxylation at position 8,9, compared to 1,2 [28]. In the hydroxylation process followed by glucuronidation, the metabolites M9-I, M9-II, and M9-III were detected with *m*/*z* values of 327.1237, 327.1435, and 327.1433, respectively, with an error of 4.1 ppm for all metabolites. The MS/MS experiments showed the fragments of a glucuronide moiety, with *m*/*z* values of 175.0237, 113.0235, and 85.0287 and the aglycon form with an *m*/*z* of 151.11. For that, M9 was tentatively identified as limonene+OH glucuronide. At least four different hydroxylated limonene metabolites have been previously reported, such as carveol (p-mentha-1,8-dien-6-ol), limonen-10-ol (p-mentha-1,8-dien-10-ol), p-mentha-1,8-dien-1-ol, and the major metabolite perillyl alcohol (p-mentha-1,8-dien-7-ol) [24,27,30]. Among these, only perillyl alcohol and limonen-10-ol were previously described as being conjugated with glucuronide [22,26,29]. The presence of a hydroxylated limonene along with a hydrogenation reaction was also detected. M16 had an *m*/*z* of 329.1585 (error 5.6 ppm) at 4.90 min and a possible MF of C_16_H_25_O_7_. The fragmentation did not show the glucuronide fragment, but for all its main fragments, such as *m*/*z* values of 113.0245, 85.0264, and 75.0080. The aglycon form was detected with an *m*/*z* of 153.1355, being tentatively identified as limonene+OH+H_2_ glucuronide. To the best of our knowledge, this is the first time this metabolite has been identified.

### 3.4. Biotransformation of D-Limonene

**Figure 3:**
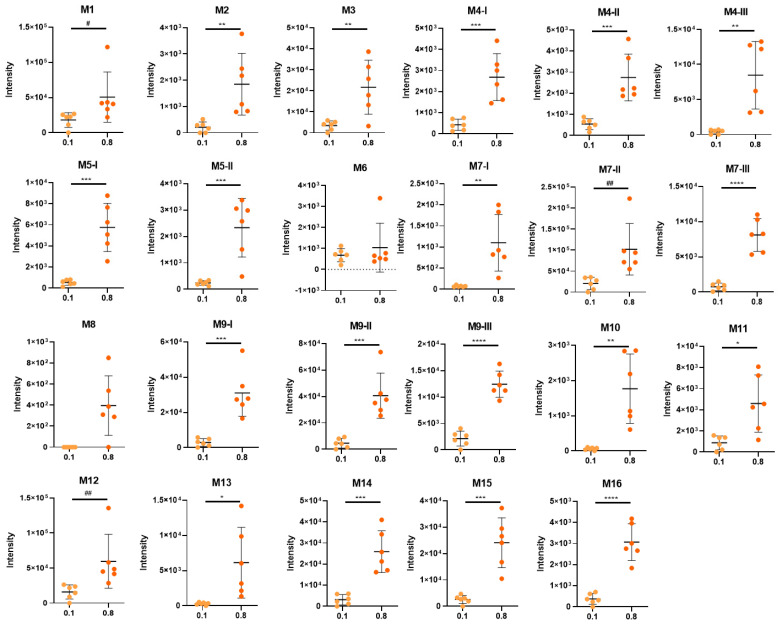
Intensity of metabolites identified in the urine of animals supplemented with 0.1% and 0.8% D-limonene expressed in mean ± SD. * Represent statistical differences using *t*-test (* *p* < 0.05; ** *p* < 0.01; *** *p* < 0.001; **** *p* < 0.0001). ^#^ Represent statistical differences using Wilcox test (^#^ *p* < 0.05; ^##^ *p* < 0.01).

Due to the high and variable production of hydroxylated metabolites, the possible route of metabolism was hydroxylation or epoxidation. Previous reports described the hydroxylation process, especially at the extremes of the molecule, at carbons C7 (perillyl alcohol) and C10 (limonene-10-ol) [24,27,30]. The epoxidation process is also described, followed by the opening of the epoxy group to produce dihydroxilated metabolites. The presence of M7 indicated that epoxidation occurs, which may be at the carbons C1-C2 (limonene-1,2-diol) or C8-C9 (uroterpenol/limonene-8,9-diol), with a preference for the latter [27,28]. Another active pathway is the carboxylation at C7, or even an oxidation of the hydroxylated metabolite at carbon C7, producing a carboxylic acid [22,23,24,25,26,27]. This process is important as it generated 13 different phase II metabolites with the carboxylic group. Hydrogenation reactions can also occur, but they generate minor metabolites, not being a first-choice pathway [25] (Figure 3 and Table 2).

**Figure 4:**
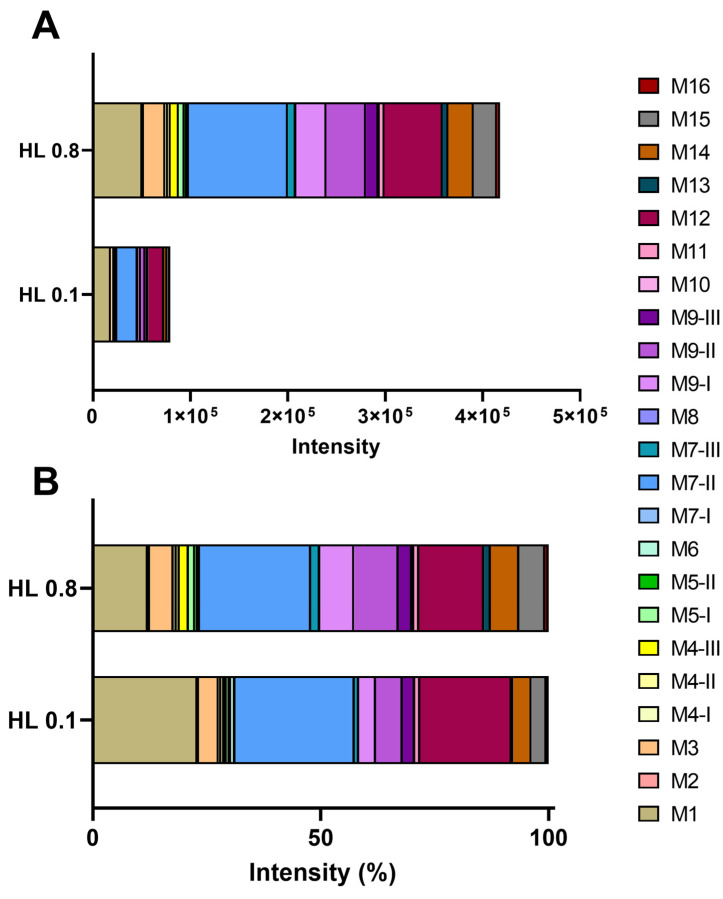
(**A**). Diversity of metabolites expressed in intensity of the chromatographic peak normalized by creatinine excretion detected in relation to the total metabolites excreted. (**B**). Diversity of metabolites expressed in percent in relation to the total metabolites excreted.

The plots of the intensity of metabolites detected in urine normalized by creatinine showed that the HL0.8 group has a greater amount of these metabolites in relation to the HL0.1 group (Figure 4). The M6 metabolite was the only one that showed no statistical difference between HL0.1 and HL0.8 and the only sulfated metabolite detected, indicating that this is not a major pathway for limonene metabolism. M8 was only detected in mice from HL0.8, suggesting that this pathway can only occur with high doses of limonene. Moreover, it is important to highlight the high variability in the intensity detected for each metabolite within the same group, even normalized by creatinine excretion, indicating high interindividual variability for limonene metabolism.

Among the identified metabolites, the most abundant are M7-II (limonene+OH+OH-glucuronide), M1 (limonene+COOH+OH+OH+glucuronide), and M12 (limonene+COOH+glycine) metabolites, which are detected at higher intensities for both groups and previously described as major products of limonene metabolization [6] (Figure 4). In the urine from the mice of the HL0.1 group, M7-II, M1, and M-12, represented 26, 23, and 20% of total metabolites, respectively. In the group HL0.8, they corresponded to 24, 12, and 14%, respectively (Figure 4, Table 2), indicating a decrease in the percentual participation of M1 and M12 in relation to the sum of all metabolites in the HL0.8 group, in comparison to HL0.1. Furthermore, the metabolites M14 (limonene+COOH+H_2_+glucuronide), M15 (limonene+COOH+H_2_+glycine), and M9-I and M9-II (limonene+OH+glucuronide) showed an increase in their percentual participation, relative to total metabolites with the higher dose of limonene. There was an increase in the percentual participation of M14, M15, M9-I, and M9II in the HL0.8 group, where they corresponded to 6.20, 5.77, 7.44, and 9.73%, respectively, in comparison to HL0.1, where they corresponded to 3.97, 3.30, 3.71, and 5.84%, respectively (Figure 4, Table 2). These results may suggest that the hydroxylation process in the limonene metabolism could be the regulating pathway, given the lower percentage of dehydroxylated metabolites (M7-II) and the appearance of only hydroxylated metabolites (M9-I and M9-II). In addition, there was a decrease in the dihydroxylation of perillic acid (M1) and an increase in metabolites with the hydrogenation reaction (M14 and M15) in HL0.8. Only metabolites M7-II, M9-II, and M14 were identified in the liver. These metabolites are at high intensities in urine, which is consistent with their detection in liver tissue.

## 4. Discussion

Following the FDA guide for conversion of animal doses to human equivalent doses, the D-limonene intake in this protocol is equivalent to a human supplementation of 7.2 mg/kg and 46.4 mg/kg for HL0.1 and HL0.8 groups, respectively [18]. Considering an adult weighing 70 kg, the daily intake would be approximately 500 mg for 0.1% and 3.25 g for 0.8%. [17]. The assessment of D-limonene intake in a population of 120 American subjects by a food frequency questionnaire focused on citrus products reported an intake range of 1.7 to 21.1 mg [19]. The limonene content in orange juice ranges from 10.7 to 520 mg/L, as described in a published review by our research group; however, it should be noted that the quantification of D-limonene in food is still uncertain and there is scarcely data regarding the intake of this compound [6].

The identification of metabolites produced by the microbiota and metabolism derived from phytochemical compounds from food sources has been indicated as responsible for the beneficial health effects [31]. Our results bring a new perspective to the metabolization of D-limonene, expanding the number of identified metabolites detected in the urine of mice. Ten new metabolites were added to the list of known metabolites [6,22,25,26,29]. The identification of three phase I molecules not previously reported to D-limonene and ten different phase II metabolites indicates new molecules with therapeutic potential to be explored in future studies [6]. Furthermore, the differences in the metabolite profile produced according to the ingested dose should be further investigated. It would be recommended to undertake studies looking into the health effects of phytochemicals or foods, considering the interindividual variability of their metabolization [6,31]. Limonene has shown potential anti-obesogenic effects by stimulating beta-oxidation in white adipose tissue and inducing browning [12,32]. In vitro studies in 3T3-L1 white adipocytes showed an induction of lipolysis by activating the gene expression of HSL and CPT1. Moreover, an increase in the expression of PRDM16, UCP1, and C/EBPβ was associated with the browning process, improved fatty acids oxidation in mitochondria, and the dissipation of the electrochemical gradient by heat production [32]. Limonene was also able to increase the uptake capacity of lipids and glucose for cellular energy expenditure, improve the adipocyte function in obese states, prevent lipotoxicity [33], and demonstrate an increase in lipolysis and glucose uptake in in 3T3-L1 cells treated with limonene and its metabolite perillyl alcohol [34]. However, to our knowledge, there are no studies with the main metabolites of limonene, such as perillic acid, perillic acid-8,9-diol, and limonene-8,9-diol, the major metabolites founded in the mice urine, as anti-obesogenic agents. For a better understanding of the mechanisms of the action of D-limonene, its metabolites should also be taken into account, since limonene is absorbed and metabolized rapidly, and its metabolites reach higher concentrations in plasma [25].

This study brought new information on the metabolism of D-limonene, revealing novel phase II metabolites. In agreement with previous reports, we also demonstrated that D-limonene may have a beneficial effect in preventing diet-induced obesity in mice. Despite the strength of the animals being individually assessed for their food intake (D-limonene consumption) and production of limonene metabolites, the relative low number of animals per group is a limitation. Follow-up studies should be performed to better characterize the metabolic changes and to investigate the mechanisms involved in the effects of D-limonene, that should be checked for correlation with the levels of limonene metabolites. Studies of the metabolization of limonene in humans are needed to translate the findings from the animal model.

## 5. Conclusions

To the best of our knowledge, in this study three new phase I metabolites, M3 (limonene+COOH+OH+H_2_+H_2_), M4 (limonene+COOH+OH+H_2_), and M16 (limonene+OH+H_2_), and ten different phase II metabolites, M2, M3, M4, M5, M6, M8, M11, M13, M15, and M16, were first attributed to D-limonene. The conjugation phase II process with glucuronic acid is the most usual, but with a higher dose of limonene conjugation with other groups, such as glycine and taurine, is observed. The metabolic switching can occur depending on the concentration of the bioactive compound supplemented. In the case of limonene, a small change occurs, decreasing hydroxylation reactions and favoring hydrogenation in phase I and conjugation with taurine and glycine in phase II. In our model, the supplementation with D-limonene at high concentrations showed no biological effects in relation to diet-induced obesity in which the lowest weight gain and fat accumulation occurred with supplementation at 0.1% D-limonene. Thus, the effect of D-limonene may not be dose dependent. Further studies are needed to confirm this phenomenon.

## Figures and Tables

**Table 1:** Characterization of essential oil rich in D-limonene.

Compound	rt	Relative Peak Area (%)	Fragments Ions ^a^
α-pinene **pin-2,3-ene**	9.12	0.57	136 (8), 121 (13), 105 (11), 93 (100), 92 (38), 91 (41), 79 (24), 77 (29), 67 (9), 53 (7)
β-phellandrene ***p*-menthane-2,7-diene**	10.43	0.75	136 (13), 121 (5), 105 (3), 93 (100), 92 (10), 91 (40), 79 (27), 77 (35), 69 (9), 53 (7)
β-pinene **pin-2,10-ene**	11.07	1.52	136 (4), 121 (5), 107 (3), 93 (100), 92 (12), 91 (22), 79 (18), 77 (16), 69 (73), 53 (12)
octanal	11.42	0.23	136 (4), 100 (16), 93 (22), 84 (72), 69 (51), 68 (38), 67 (29), 57 (100), 56 (89), 55 (78)
Δ^3^-carene **car-3,4-ene**	11.56	0.14	136 (18), 121 (19), 105 (14), 93 (100), 92 (24), 91 (42), 80 (24), 79 (34), 77 (38), 67 (11)
*m*-cymene ***m*-menthane-1,3,5-triene**	12.03	0.01	134 (22), 119 (100), 117 (16), 115 (8), 103 (2), 91 (24), 77 (11), 65 (8), 51 (6), 56 (6)
limonene ***p*-mentha-1,8-diene**	12.26	96.45	136 (21), 121 (24), 107 (23), 93 (73), 92 (24), 91 (22), 79 (36), 67 (76), 68 (100), 53 (23)
γ-terpinene ***p*-menthane-1,4-diene**	13.04	0.07	136 (36), 121 (30), 119 (14), 105 (11), 93 (100), 92 (26), 91 (55), 79 (27), 77 (36), 53 (9)
linalool **3,7-dimethylocta1,6-dien-3-ol**	14.21	0.27	154 (6), 136 (8), 121 (22), 105 (7), 93 (78), 83 (17), 80 (32), 71 (100), 69 (44), 55 (58)

rt (retention time); ^a^ *m*/*z* fragments expressed in relative abundance. Bold names use the nomenclature suggested by Grafflin (1955) for American Chemical Society.

**Table 2:** Identification of D-limonene metabolites in urine and liver samples after a six-week intake.

ID	Metabolite	rt	[M-H]^−^	MS/MS	Error	[MF]-H	HL0.1%	HL0.8%	T
M1	limonene+COOH+OH+OH glucuronide **dihydroxy-*p*-mentha-1,8-dien-7-oic acid glucuronide** *perillic acid-8,9-diol glucuronide*	1.89	375.1304	375.1333 (20); 357.1104 (5); 183.1088 (5); 172.9903 (100); 160.0421 (15); 113.0224 (40); 93.0353 (85); 85.0291 (45); 75.0086 (65); 59.0141 (75)	−1.8	C_16_H_23_O_10_	23.00	12.13	U
M2	limonene+COOH+OH+OH+H_2_ glucuronide **dihydroxy-*p*-mentha-1-en-7-oic acid/dihydroxy-*p*-mentha-8-en-7-oic acid glucuronide**	2.01	377.1460	377.1469 (100); 204.0368 (10); 201.1180 (5); 178.0497 (20); 175.0236 (5); 172.9923 (60); 160.0399 (33); 113.0251 (30); 93.0344 (55); 85.0293 (35); 75.0087 (60); 71.0145 (70); 59.0147 (70)	−1.9	C_16_H_25_O_10_	0.27	0.44	U
M3	limonene+COOH+OH+H_2_+H_2_ glucuronide **dihydroxy-*p*-menthan-7-oic acid glucuronide**	2.83	361.1504	361.1500 (85); 301.1299 (5); 185.1181 (10); 175.0256 (3) 157.0128 (5); 113.0239 (60); 85.0294 (65); 71.0138 (90); 59.0141 (100)	−0.1	C_16_H_25_O_9_	4.37	5.18	U
M4-I	limonene+COOH+OH+H_2_ glucuronide I **hydroxy-*p*-menth-8-en-7-oic acid glucuronide**	2.95	359.1344	359.1347 (35); 297.1331 (5); 241.1095 (5); 183.1047 (15); 175.0242 (5); 113.0241 (45); 99.0084 (15); 85.0290 (60); 75.0088 (100); 71.0131 (80); 59.0143 (95); 44.9989 (20)	1.0	C_16_H_23_O_9_	0.54	0.64	U
M4-II	limonene+COOH+OH+H_2_ glucuronide II **hydroxy-*p*-menth-8-en-7-oic acid glucuronide**	3.18	359.1345	359.1332 (10);315.1646 (5); 183.1020 (65); 175.0240 (5); 155.1070 (25); 137.0971 (10); 113.0249 (30); 99.0097 (10); 85.0294 (40); 71.0137 (45); 59.0137 (100); 44.9985 (20)	0.8	C_16_H_23_O_9_	0.68	0.66	U
M5-I	limonene+COOH+OH+H_2_ glycine I **hydroxy-*p*-menth-8-en-7-oic acid glycine**	3.41	240.1224	240.1228 (5); 178.1213 (1); 111.0801 (5); 74.0242 (100); 72.0453 (20); 56.0152 (2)	7.2	C_12_H_18_NO_4_	0.68	1.38	U
M4-III	limonene+COOH+OH+H_2_ glucuronide III **hydroxy-*p*-menth-8-en-7-oic acid glucuronide**	3.57	359.1326	183.1015 (100); 175.0243 (5); 137.0965 (30); 113.0233 (45); 99.0080 (15); 85.0292 (35); 71.0137 (30); 59.0138 (80); 44.9983 (30)	6.0	C_16_H_23_O_9_	0.50	2.02	U
M6	limonene+COOH sulfate ***p*-mentha-1,8-dien-7-oic acid sulfate** *perillic acid sulfate*	3.62	245.0490	245.0517 (20); 165.0919 (10); 79.9569 (20); 74.0241 (20); 59.0151 (45)	−0.3	C_10_H_13_O_5_S	0.85	0.25	U
M5-II	limonene+COOH+OH+H_2_ glycine II **hydroxy-*p*-menth-8-en-7-oic acid glycine**	3.62	240.1229	240.1229 (5); 178.1244 (1); 111.0793 (5); 74.0244 (100); 72.0446 (20); 56.0148 (2)	5.3	C_12_H_18_NO_4_	0.30	0.56	U
M7-I	limonene+OH+OH glucuronide I ***p*-menth-8-en-1,2-diol/*p*-menth-1-en-8,9-diol glucuronide** *limonene-1,2-diol*/*limonene-8,9-diol (uroterpenol) glucuronide*	3.71	345.1550	345.1546 (50); 327.1414 (5); 285.1317 (5); 269.1388 (5); 193.0382 (5); 175.0227 (5); 169.1228 (5); 157.0139 (10); 113.0236 (60); 99.0082 (10); 85.0291 (70); 75.0084 (100); 59.0138 (60)	1.4	C_16_H_25_O_8_	0.09	0.26	U
M7-II	limonene+OH+OH glucuronide II ***p*-menth-8-en-1,2-diol/*p*-menth-1-en-8,9-diol glucuronide** *limonene-1,2-diol*/*limonene-8,9-diol (uroterpenol) glucuronide*	3.80	345.1548	345.1537 (50); 327.1414 (5); 285.1324 (5); 269.1388 (5); 193.0382 (5); 175.0227 (5); 169.1207 (5); 157.0133 (10); 113.0237 (60); 99.0082 (10); 85.0291 (70); 75.0084 (100); 59.0141 (60)	2.1	C_16_H_25_O_8_	26.15	24.36	U, L
M8	limonene+COOH+OH+H_2_ taurine I **hydroxy-*p*-menth-8-en-7-oic acid taurine**	3.92	290.1054	290.2051 (45); 152.0006 (10); 124.0063 (30); 106.9814 (10); 79.9570 (100); 74.0242 (85)	4.6	C_12_H_20_NO_5_S	0.00	0.09	U
M7-III	limonene+OH+OH glucuronide III ***p*-menth-8-en-1,2-diol/*p*-menth-1-en-8,9-diol glucuronide** *limonene-1,2-diol*/*limonene-8,9-diol (uroterpenol) glucuronide*	4.13	345.1538	n.d.	5.0	C_16_H_25_O_8_	0.95	1.94	U
M9-I	limonene+OH glucuronide I ***p*-mentha-1,8-dien-1-ol/*p*-mentha-1,8-dien-6-ol/*p*-mentha-1,8-dien-7-ol/*p*-mentha-1,8-dien-10-ol glucuronide** p*-mentha-1,8-dien-1-ol*/*carveol*/*perillyl alcohol*/*limonene-10-ol glucuronide*	4.36	327.1437	327.1439 (20); 309.1323 (5); 209.1191 (5); 175.0247 (3); 157.0141 (5); 151.1132 (2); 113.0233 (30); 85.0290 (60); 75.0085 (100); 59.0139 (80)	4.1	C_16_H_23_O_7_	3.71	7.44	U
M10	limonene+COOH glucuronide ***p*-mentha-1,8-dien-7-oic acid glucuronide** *perillic acid glucuronide*	4.49	341.1243	261.0763 (10); 175.0230 (3); 165.0923 (45); 113.0241 (35); 99.0081 (20); 85.0295 (55); 71.0141 (40); 59.0142 (100); 44.9993 (25)	−0.3	C_16_H_21_O_8_	0.08	0.42	U
M9-II	limonene+OH glucuronide II ***p*-mentha-1,8-dien-1-ol/*p*-mentha-1,8-dien-6-ol/*p*-mentha-1,8-dien-7-ol/*p*-mentha-1,8-dien-10-ol glucuronide** p*-mentha-1,8-dien-1-ol*/*carveol*/*perillyl alcohol*/*limonene-10-ol glucuronide*	4.51	327.1435	327.1424 (15); 209.1177 (5); 175.0237 (1); 157.0123 (5); 151.1120 (1); 113.0235 (30); 85.0287 (10); 75.0083 (100); 57.0348 (45); 44.9985 (20)	4.1	C_16_H_23_O_7_	5.84	9.73	U, L
M11	limonene+COOH+H_2_ taurine ***p*-menth-8-en-7-oic acid/*p*-menth-1-en-7-oic acid taurine** *dihydroperillic acid taurine*	4.55	274.1101	274.1092 (60); 206.0483 (5); 124.0062 (5); 106.9791 (10); 79.9570 (100); 72.0445 (5)	6.2	C_12_H_20_NO_4_S	1.10	1.10	U
M12	limonene+COOH glycine ***p*-mentha-1,8-dien-7-oic acid glycine** *perillic acid glycine*	4.63	222.1134	222.1120 (25); 178.1218 (75); 176.1078 (80); 121.1019 (25); 119.0852 (45); 110.0612 (35); 108.0453 (20); 74.0246 (100); 56.0143 (15)	0.8	C_12_H_16_NO_3_	20.04	14.26	U
M13	limonene+COOH taurine ***p*-mentha-1,8-dien-7-oic acid taurine** *perillic acid taurine*	4.66	272.0949	272.0949 (35); 204.0322 (25); 164.1039 (3); 124.0070 (5); 106.9803 (10); 79.9567 (100)	5	C_12_H_18_NO_4_S	0.38	1.47	U
M9-III	limonene+OH glucuronide III ***p*-mentha-1,8-dien-1-ol/*p*-mentha-1,8-dien-6-ol/*p*-mentha-1,8-dien-7-ol/*p*-mentha-1,8-dien-10-ol glucuronide** p*-mentha-1,8-dien-1-ol*/*carveol*/*perillyl alcohol*/*limonene-10-ol glucuronide*	4.71	327.1433	327.1429 (15); 309.1361 (3); 209.1166 (5); 151.1120 (15); 113.0235 (10); 85.0289 (50); 83.0498 (65); 75.0083 (100); 57.0348 (35); 44.9984 (10)	4.1	C_16_H_23_O_7_	2.69	2.98	U
M14	limonene+COOH+H_2_ glucuronide ***p*-menth-8-en-7-oic acid/*p*-menth-1-en-7-oic acid glucuronide** *dihydroperillic acid glucuronide*	4.71	343.1389	175.0235 (3); 167.1069 (75); 113.0238 (40); 99.0081 (20); 85.0288 (50); 71.0133 (35); 59.0139 (100)	1.3	C_16_H_23_O_8_	3.97	6.20	U, L
M15	limonene+COOH+H_2_ glycine ***p*-menth-8-en-7-oic acid/*p*-menth-1-en-7-oic acid glycine** *dihydroperillic acid glycine*	4.71	224.1277	224.1287 (5); 180.1399 (5); 178.1203 (10); 54.0479 (5); 74.0247 (100); 56.0151 (5)	6.7	C_12_H_18_NO_3_	3.30	5.77	U
M16	limonene+OH+H_2_ glucuronide ***p*-mentha-1-en-1-ol/*p*-mentha-1-en-6-ol/*p*-mentha-1-en-7-ol/*p*-mentha-1-en-10-ol/*p*-mentha-8-en-1-ol/*p*-mentha-8-en-6-ol/*p*-mentha-8-en-7-ol/*p*-mentha-8-en-10-ol glucuronide**	4.90	329.1585	329.1575 (15); 269.1356 (5); 211.1321 (5); 153.1355 (3); 113.0245 (20); 85.0264 (65); 75.0080 (100); 55.0186 (35)	5.6	C_16_H_25_O_7_	0.47	0.73	U

rt (retention time); [M-H]^−^ (experimental *m*/*z* value in negative charge); MS/MS (*m*/*z* value of fragmentation experiments expressed in relative abundance); MF (molecular formula); n.d. (not detected); error (ppm); HL0.1% (percent of urine metabolite area in relation to all metabolites area for HL0.1 group); HL0.8% (percent of urine metabolite area in relation to all metabolites area for HL0.8 group); T (tissue); U (urine); L (liver). Bold names use the nomenclature suggested by Grafflin (1955) for American Chemical Society. Italic names use the literature nomenclature.

## Data Availability

The data presented in this study are available on request from the corresponding author. The data are not publicly available due to ongoing research.

## Supplementary Materials

The following supporting information can be downloaded at: https://www.mdpi.com/article/10.3390/metabo12121246/s1, Figure S1: Weight gain, energy efficiency, limonene intake and tissues weight of animals supplemented with 0.1 and 0.8% D-limonene. Weight gain over six weeks of D-limonene supplementation (**A**); Food intake over six weeks of D-limonene supplementation (**B**); Weight gain (**C**); energy efficiency (**D**); limonene intake (**E**); Liver weight (**F**); Epididymal adipose tissue (EAT) weight (**G**); Retroperitonial adipose tissue (RAT) weight (**H**) and subcutaneous adipose tissue (SAT) weight (**I**). * Represent statistical differences using ANOVA Tukey test (* *p* < 0.05; ** *p* < 0.01; *** *p* < 0.001. ^#^ Represent statistical differences using Kruskal–Wallis test (^#^ *p* < 0.05; ^###^ *p* < 0.001). For Figure S1A * Represent statistical differences using two-way ANOVA (* *p* < 0.05).
