# Supplementary figures and images for: Identification of D-Limonene Metabolites by LC-HRMS: An Exploratory Metabolic Switching Approach in a Mouse Model of Diet-Induced Obesity
Source: Metabolites. 2022 Dec 9;12(12):1246. doi: 10.3390/metabo12121246 (PMC9780935; doi:10.3390/metabo12121246)

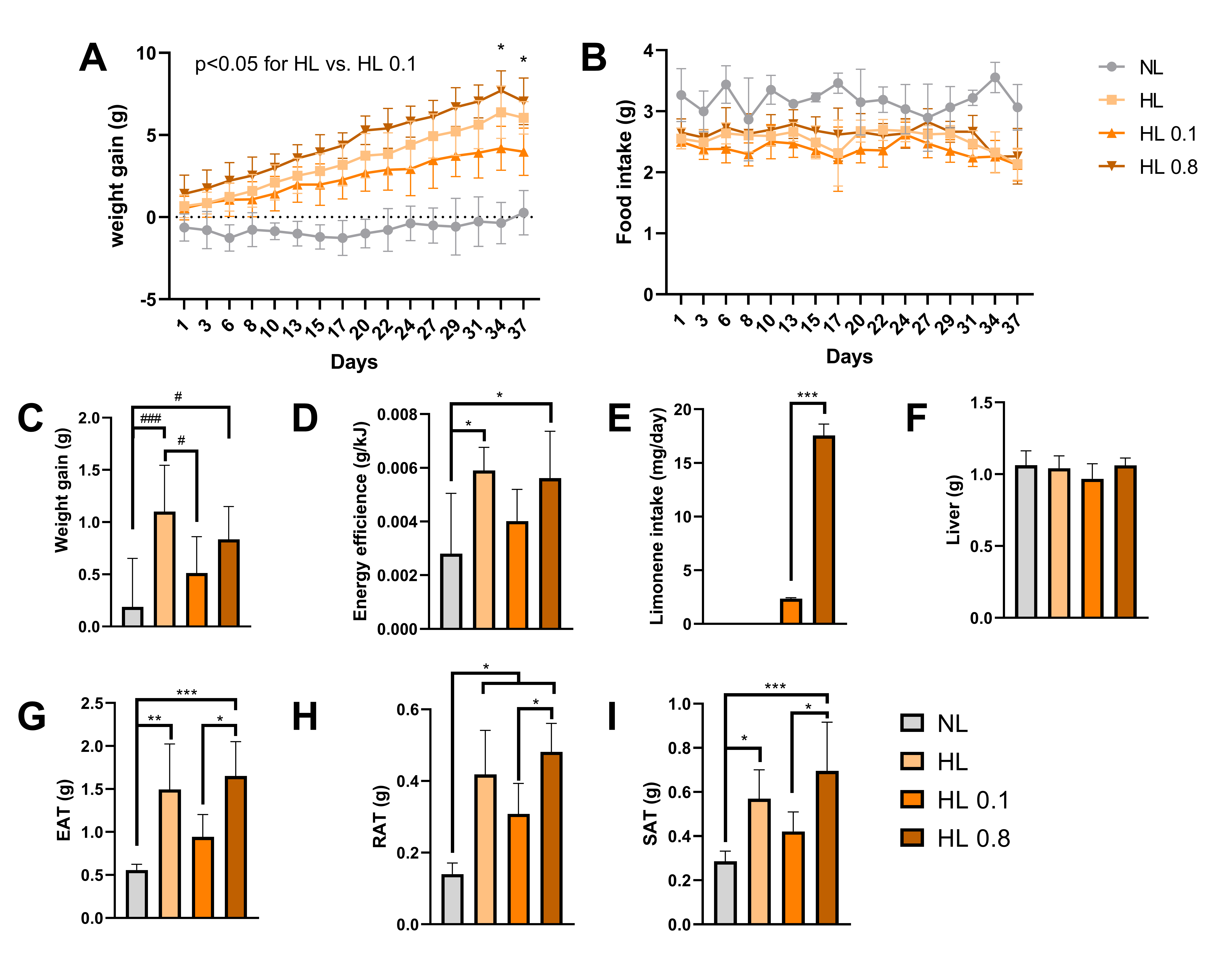

Supplement: Supplementary file 1 [file metabolites-12-01246-s001.zip › metabolites-2055661-supplementary.png]
